# Combination of Rhamnetin and RXP03 Mitigates Venom-Induced Toxicity in Murine Models: Preclinical Insights into Dual-Target Antivenom Therapy

**DOI:** 10.3390/toxins17060280

**Published:** 2025-06-04

**Authors:** Jianqi Zhao, Guangyao Liu, Xiao Shi, Chunhong Huang

**Affiliations:** 1 School of Basic Medical Sciences, Jiangxi Medical College, Nanchang University, Nanchang 30006, Jiangxi, China; 2 The First Clinical Medical College, Nanchang University, Nanchang 330047, China; 3 Queen Mary School, Jiangxi Medical College, Nanchang University, Nanchang 330006, Jiangxi, China

**Keywords:** snake venom, small molecule inhibitors, antivenom drugs, Rhamnetin, RXP03

## Abstract

Snakebite is a significant global public health challenge, and the limited application of antivenom has driven the exploration of novel therapies. Combination therapy using small-molecule drugs targeting phospholipases A_2_ (PLA2) and metalloproteinases (SVMP) in venom shows great potential. Although Rhamnetin and RXP03 exhibit notable anti-phospholipase and anti-metalloproteinase activities, respectively, their antiophidic potential remains poorly explored. This study aims to evaluate the inhibitory effects of Rhamnetin and RXP03 on snake venom toxicity. Methodologically, we conducted in vitro enzymatic assays to quantify PLA_2_/SVMP inhibition, murine models of envenomation (subcutaneous/intramuscular venom injection) to assess local tissue damage and systemic toxicity, and histopathological/biochemical analyses. In vitro experiments demonstrated that Rhamnetin effectively inhibited PLA_2_ activity while RXP03 showed potent suppression of SVMP activity, with their combination significantly reducing venom-induced hemorrhagic activity. In murine models, the combined therapy markedly alleviated venom-triggered muscle toxicity and ameliorated oxidative stress. Furthermore, the combination enhanced motor performance and survival rate in mice, improved serum biochemical parameters, corrected coagulation disorders, and attenuated pathological damage in liver, kidney, heart, and lung tissues. This research demonstrates that dual-targeted therapy against metalloproteinases and phospholipases in snake venom can effectively prevent a series of injuries caused by snake venom. Collectively, the combined application of Rhamnetin and RXP03 exhibits significant inhibitory effects on a variety of venom-induced toxicities, providing pharmacological evidence for the development of antivenom therapies. However, the efficacy validation in this study was limited to murine models, and there is a discrepancy with clinical needs for delayed treatment in real-world envenomation scenarios. Despite these limitations, the findings provide robust preclinical evidence supporting the Rhamnetin–RXP03 combination therapy as a cost-effective, broad-spectrum antivenom strategy. Future studies are required to optimize dosing regimens and evaluate clinical translatability.

## 1. Introduction

Snakebite constitutes a major challenge to global public health systems, particularly posing severe health threats to low-income populations in tropical regions [1,2]. The World Health Organization (WHO) classified snakebite as a “Category A Neglected Tropical Disease” in 2017 and aims to reduce related mortality by 50% by 2030 [3,4]. Although antivenom remains the only specific therapy currently available and is listed on the WHO Essential Medicines List, its application is constrained by critical limitations including narrow species specificity, complex production processes (reliant on equine immunization), high cold-chain storage and transportation costs, and potential allergic risks [5,6,7,8]. Furthermore, as most snakebites occur in economically disadvantaged areas, the requirement for intravenous administration by trained medical professionals severely restricts accessibility in remote regions [1,9]. Therefore, the development of novel therapeutic approaches has become an urgent imperative.

In recent years, small-molecule inhibitors have emerged as a promising direction in antivenom drug development due to their advantages of high chemical stability and cost-effective large-scale production [10,11,12,13,14,15]. These agents block pathological effects by targeting key toxic proteins in venom and can achieve cross-species coverage through multi-target combinations [13,15,16]. For example, the combined use of phospholipase A2 (PLA_2_) inhibitor varespladib and snake venom metalloprotease (SVMP) inhibitor marimastat has been shown to significantly reduce mortality in mice injected with *Deinagkistrodon acutus* venom [10,17]. Notably, PLA_2_ and SVMP constitute major components in Viperidae and Elapidae venoms and are directly associated with core symptoms such as hemorrhage and necrosis [18,19,20,21,22,23]. Taking common Chinese venomous snakes as examples: SVMP accounts for approximately 30% of the venom composition in *Deinagkistrodon acutus* (*D. acutus*, Viperidae) and *Protobothrops mucrosquamatus* (*P. mucrosquamatus*, Viperidae) [20,22], while reaching up to 40% in *Gloydius blomhoffi siniticus* (*G. blomhoffi*, Viperidae) [22]. PLA_2_ also exceeds 30% in venoms of *Naja atra* (*N. atra*, Elapidae) and *Trimeresurus stejnegeri* (*T. stejnegeri*, Viperidae) [20,24]. Previous studies have confirmed that SVMP inhibitors alone can mitigate viperid snake venom-induced myotoxicity [25], while varespladib monotherapy alleviates *N. atra*-caused hepatic injury [26]. Therefore, developing inhibitors targeting PLA_2_ and SVMP holds significant clinical importance.

Rhamnetin (molecular formula: C16H12O7), a flavonoid compound derived from coriander, is characterized by four hydroxyl (OH) groups in its structure. It exhibits antioxidant, anti-inflammatory, and antiviral activities, and has been shown to inhibit secretory PLA_2_ activity [27]. Experimental studies have demonstrated that Rhamnetin significantly suppresses PLA_2_ activity in Bothrops jararacussu venom, reducing paw edema volume and markedly decreasing plasma creatine kinase (CK) levels in mice [28]. However, the inhibitory effects of Rhamnetin on PLA_2_ in other snake venoms remain unclear. RXP03, an inhibitor of matrix metalloproteinases (MMPs), demonstrates potent activity against various metalloproteinases (MMP-2, MMP-8, MMP-9, MMP-11, MMP-14) due to its unique structural properties [29,30], though its inhibitory effects on SVMP have not been confirmed yet.

In this study, we conducted in vitro enzymatic activity assays to evaluate the inhibitory effects of Rhamnetin on PLA_2_ and RXP03 on SVMP activity. In animal experiments, the combination of these two agents was administered to mice, followed by comprehensive assessments of muscular toxicity, hematological and biochemical parameters, and organ functions. The research demonstrated that these two drugs can significantly inhibit snake venom-induced muscular toxicity and systemic injuries in mice, while reducing mortality rates. This provides novel pharmacological evidence for the development of antivenom therapeutics.

## 2. Results

### 2.1. Inhibitory Effect of Venom

**Figure 1:**
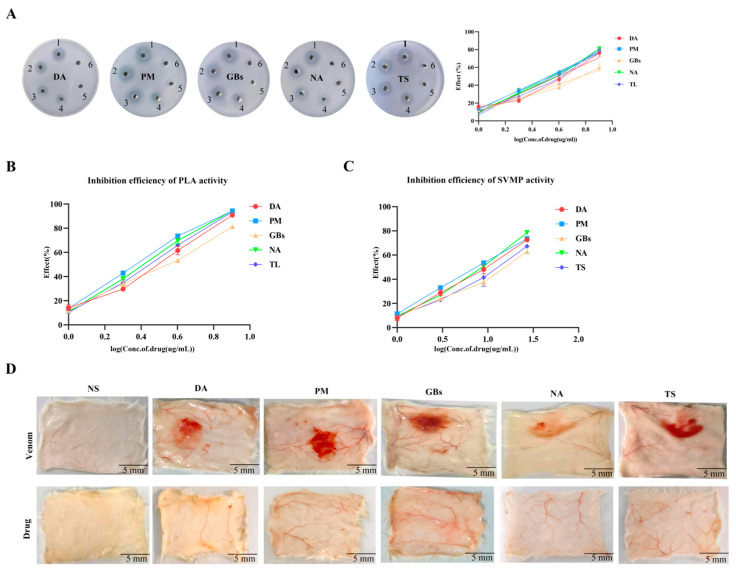
Inhibition of venom enzymatic activities and associated biological effects by Rhamnetin and RXP03. (**A**) The action performance of different snake venoms (DA, PM, GBs, NA, TS) on the plate and the inhibitory efficiency. Well 1 was filled with venom. Wells 2 to 5 were mixtures of venom and different concentrations of Rhamnetin. Well 6 was the negative control. (**B**) Test the inhibitory efficiency of Rhamnetin on the activity of PLA_2_ using NOB as the substrate. (**C**) The inhibitory efficiency of RXP03 on the activity of SVMP. (**D**) Anti-venom hemorrhagic activity of the combination of Rhamnetin and RXP03. Error bars represent the standard error of the mean for *n* = 3 replicates.

The egg yolk plate assay results demonstrated that Rhamnetin exhibited inhibitory effects on the PLA_2_ activity of all five snake venoms tested (Figure 1A). Rhamnetin has inhibitory effects on PLA_2_ in venom. However, the inhibition increased proportionally with higher drug-to-venom ratios. As illustrated in Figure 1C, RXP03 demonstrated a significant inhibitory effect on SVMP activity in the venoms. Combined use of two drugs was able to decrease the hemorrhagic effect caused by snake venoms (Figure 1D).

### 2.2. Rhamnetin and RXP03 Mitigated Venom-Induced Myotoxic Activity

**Figure 2:**
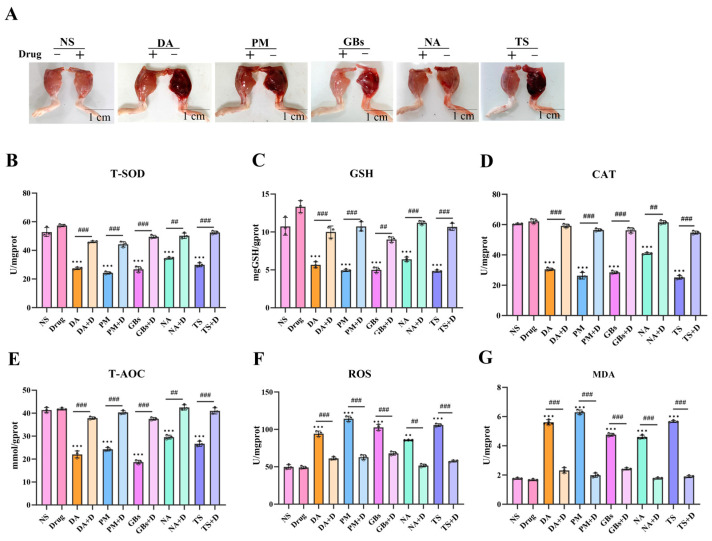
Alleviation of venom-induced oxidative stress and muscle function impairment. (**A**) Snake venom-induced muscle hemorrhage and edema were significantly alleviated by drug treatment. The muscle’s ability to resist tension has returned to normal. (**B**–**G**) Inhibitory effect of Rhamnetin and RXP03 in venom-induced serum oxidative stress. Antioxidant capacity (T-SOD, GSH, CAT, T-AOC) recovered and oxidative products of muscle (ROS, MDA) were reduced under the intervention of drugs. Data shown as mean ± standard error of the mean (*n* = 3). * represents significantly different from the NS group; #, venom group vs. drug-treated group (** *p* ≤ 0.01, *** *p* ≤ 0.001, ## *p* ≤ 0.01, and ### *p* ≤ 0.001).

As shown in Figure 2, the snake venoms caused significant damage to mouse muscle tissue, while drug treatment significantly reduced the degree of muscle injury, consistent with the data obtained from muscle tension tests. In terms of antioxidant indicators, compared to the saline group, the venom-treated groups exhibited altered levels of multiple antioxidant enzymes (e.g., T-SOD, GSH, CAT, and T-AOC), along with increased levels of ROS and MDA. Drug intervention reversed the oxidative stress damage induced by the venoms. Furthermore, when the drugs were administered alone, the aforementioned antioxidant-related parameters in muscle tissue showed no significant changes.

**Figure 3:**
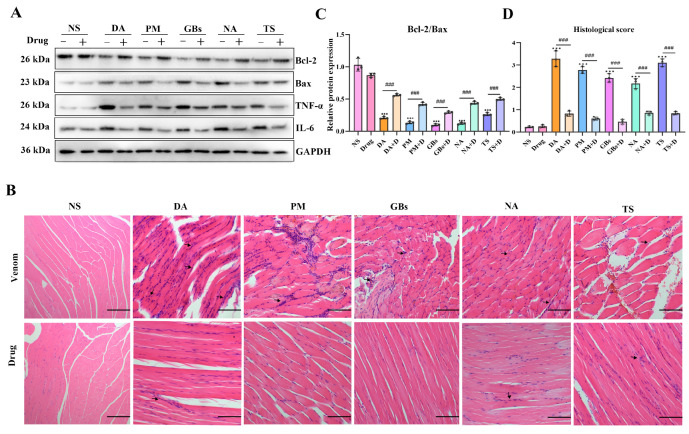
Inflammatory mediators-related protein expression and histopathological changes in muscle tissue. (**A**,**C**) Western blotting of Bcl-2, Bax, TNF-α, and IL-6 in gastrocnemius muscle and quantitative analysis of the expressions of Bax and Bcl-2. (**B**) Hematoxylin and eosin staining of gastrocnemius muscle sections. Black arrows indicate inflammatory cell infiltration. Scale bar, 200 μm. (**D**) Muscle section pathological scoring. The results are shown as mean ± standard error of the mean (*n* = 3). * represents significantly different from the NS group; #, venom group vs. drug-treated group (*** *p* ≤ 0.001 and ### *p* ≤ 0.001).

As illustrated in Figure 3A, the snake venoms increased the levels of muscle inflammatory proteins (TNF-α, IL-6) and the pro-apoptotic protein Bax, while decreasing the anti-apoptotic protein Bcl-2. Drug treatment induced a significant recovery trend in the expression of these proteins. Histopathological analysis revealed that (Figure 3B), compared to the saline group, the venom-treated groups exhibited marked pathological alterations in muscle tissue structure, including abnormal cellular morphology, disorganized fiber arrangement, and inflammatory cell infiltration. Necrotic areas were observed in the PM and GBs groups. Drug intervention effectively alleviated venom-induced muscle tissue damage, restoring the structural organization toward normalcy and reducing histological scores. However, the drugs did not completely reverse the tissue damage caused by the venoms. Compared to the NS group, the DA+D group showed slightly disorganized muscle fiber arrangement, while the NA+D and TS+D groups displayed mildly abnormal muscle fiber morphology and nuclear centralization, potentially suggesting ongoing inflammatory responses or tissue repair processes. Additionally, when the drugs were administered alone, no significant changes in protein expression or pathological structure were observed in mouse muscle tissue.

### 2.3. Rhamnetin and RXP03 Ablated Systemic Toxicity Induced by Venom

**Figure 4:**
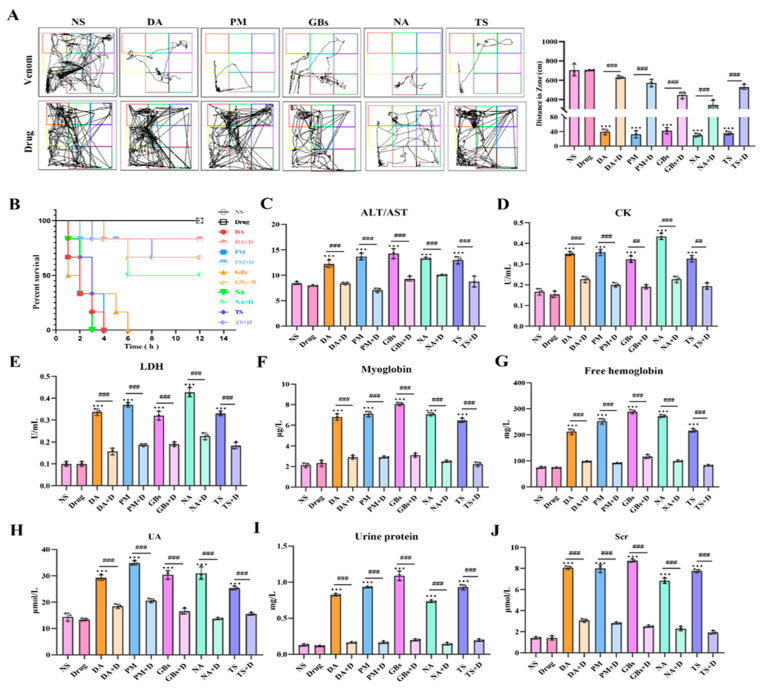
Inhibition of systemic toxicity of snake venom by Rhamnetin and RXP03. (**A**) Motor function trajectories of mice 12 h post-envenoming. Venom groups (DA, PM, GBs, NA, TS) show restricted movement vs. saline control (NS). (**B**) Kaplan–Meier survival curves over 24 h. Venom-only groups exhibited rapid lethality (20% survival), while drug-treated groups showed significant protection (85% survival, *p* < 0.001). (**C**–**H**,**J**) Serum biochemical markers: Drug intervention normalized venom-elevated levels of (**C**) ALT/AST (liver damage), (**D**) CK (myotoxicity), (**E**) LDH (systemic injury), (**F**,**G**) myoglobin/free hemoglobin (rhabdomyolysis), (**H**,**J**) UA/Scr (renal impairment). (**I**) Urinary protein. * represents significantly different from the NS group; #, venom group vs. drug-treated group (*** *p* ≤ 0.001, ## *p* ≤ 0.01, and ### *p* ≤ 0.001).

In terms of locomotor ability in mice (Figure 4A), snake venom treatment significantly impaired the motor ability of mice, affecting both their movement trajectories and total distance traveled. Compared to the NS group, the venom-treated groups exhibited markedly shortened movement trajectories. However, drug treatment led to varying degrees of improvement in motor function across all venom-treated groups. This difference arose from variations in venom composition and toxic mechanisms. Regarding survival outcomes, the survival rate of venom-treated mice rapidly declined over time, with the rate and extent of decline differing among venom groups. In contrast, the survival rate of drug-treated groups was significantly higher than that of venom-only groups, demonstrating the drugs’ efficacy in mitigating venom-induced lethality (Figure 4B). In terms of biochemical parameters (Figure 4C–J), compared to the saline group, venom-treated mice showed significant alterations in multiple serum markers. Levels of ALT/AST, CK, LDH, myoglobin, free hemoglobin, UA, Scr, and urinary protein were significantly elevated, reflecting venom-induced damage to the liver, muscles, kidneys, and disruption of normal physiological metabolism. Following drug intervention, these indicators showed a notable downward trend, indicating that the drugs effectively alleviated venom-triggered systemic damage.

### 2.4. Rhamnetin and RXP03 Prevented Venom-Induced Hemostatic Dysfunction

**Figure 5:**
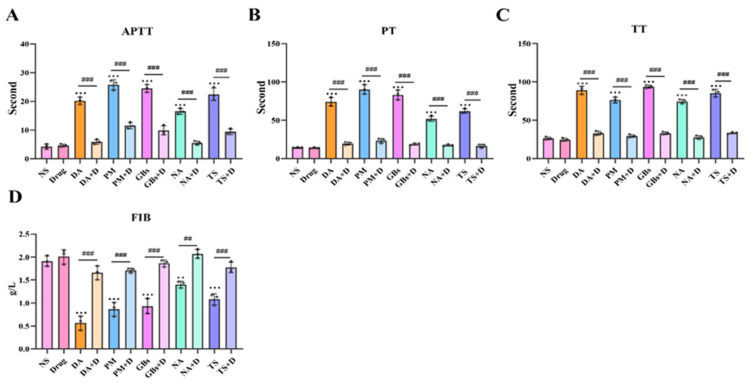
Rhamnetin and RXP03 prevention of venom-induced coagulopathy. Under the influence of venom, the coagulation function is disrupted and APTT (**A**), PT (**B**), and TT (**C**) significantly increase. The snake venom depletes FIB (**D**) in the blood of the poisoned mice. Drugs reverse this effect. The results were expressed as mean ± standard deviation (*n* = 3). * represents significantly different from the NS group; #, venom group vs. drug-treated group (** *p* ≤ 0.01, *** *p* ≤ 0.001, ## *p* ≤ 0.01, and ### *p* ≤ 0.001).

In terms of coagulation parameters (Figure 5), compared to the NS group, APTT, PT, and TT were significantly prolonged and FIB levels were significantly reduced after venom treatment, indicating that the venoms interfered with both the intrinsic and extrinsic coagulation pathways as well as fibrinogen levels, severely impairing normal coagulation function. Following drug intervention, these parameters showed a marked recovery trend, demonstrating that the drugs effectively reversed the adverse effects of the venoms on coagulation function.

### 2.5. Rhamnetin and RXP03 Attenuated Histopathological Destruction Caused by Venom

**Figure 6:**
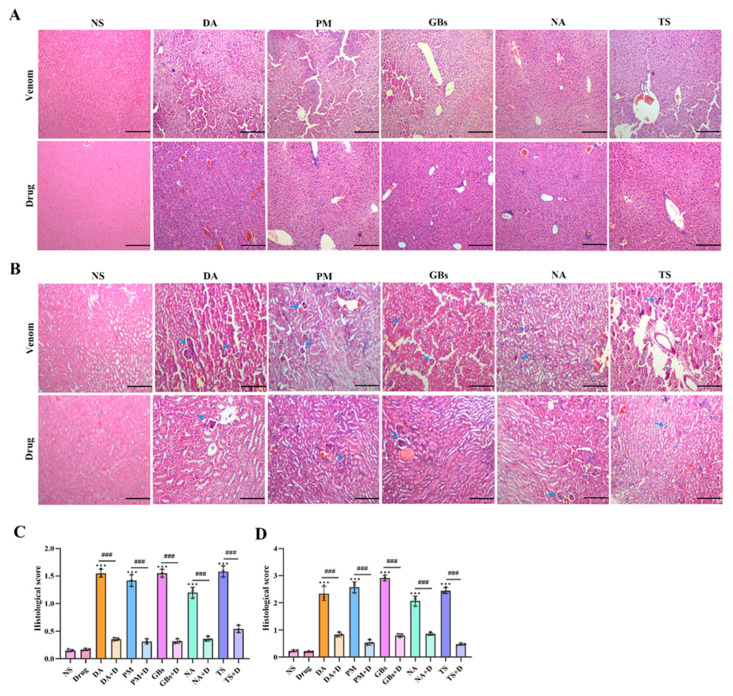
Hematoxylin–eosin staining of liver and renal tissue. Venom-induced severe liver and kidney damage occurred. The results indicated hepatocyte necrosis and inflammatory infiltrate (**A**). In the renal tissue the glomeruli are contracted (blue arrow), and the structure of the renal tubules shows abnormality (**B**). The pathological damage degrees of liver and kidney have significantly decreased after drug intervention. (**C**) Liver histopathology scores. (**D**) Renal histopathology scores. Scale bar, 200 μm. Data shown as mean ± standard deviation (*n* = 3). * represents significantly different from the NS group; #, venom group vs. drug-treated group (*** *p* ≤ 0.001 and ### *p* ≤ 0.001).

In acute snake venom poisoning events, severe liver and kidney damage is commonly observed [31]. As shown in Figure 6A, H&E staining results of liver tissue sections revealed that, compared with the normal saline group (NS), the snake venom-treated groups (DA, PM, etc.) exhibited hepatocyte necrosis, inflammatory cell infiltration, and blurred cell boundaries, indicating that the venom caused significant damage to liver tissue. After drug intervention, inflammatory cell infiltration was markedly reduced, the liver tissue structure became intact and clear, and cell morphology appeared more regular, with pathological damage substantially alleviated. Kidney tissue staining results demonstrated that snake venom treatment induced glomerular swelling, extensive inflammatory cell infiltration, and renal interstitial congestion (Figure 6B). In contrast, the drug-treated groups displayed well-defined and intact glomerular morphology and renal tubular structures. The histological staining results of kidney tissue indicated that drug treatment significantly reduced liver and kidney injury scores. These findings collectively demonstrated that the drug effectively mitigated both physiological and pathological damage to liver and kidney tissues caused by snake venom (Figure 6C,D). This provides a histopathological explanation for the improvement in related biochemical indicators (e.g., ALT, AST, UA, SCr) observed after drug treatment.

**Figure 7:**
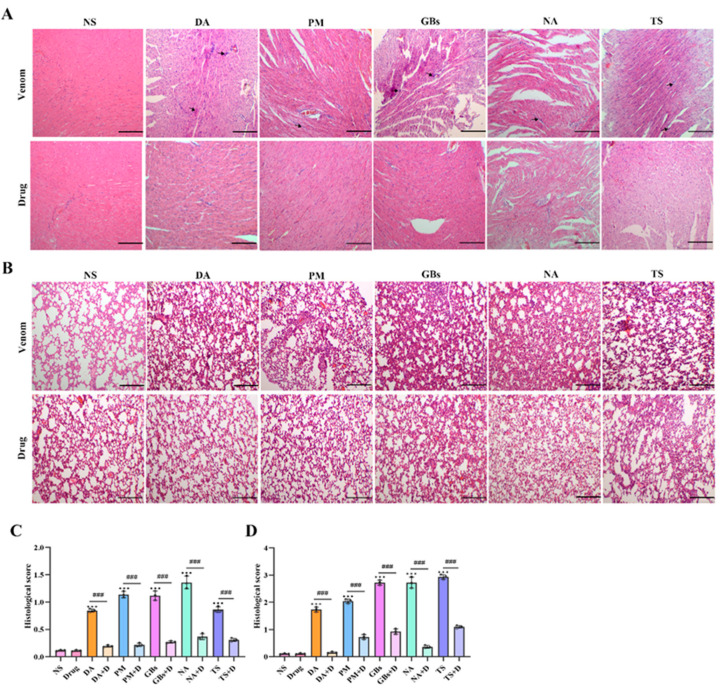
Hematoxylin–eosin staining of cardiac and lung tissues. Snake venom exposure led to substantial damage in both cardiac and lung tissues. In the cardiac tissue (**A**), disruptions in the tissue structure, inflammatory infiltrate, and cell damage were observed in the venom-treated groups. In the lung tissue (**B**), there is obvious blood infiltration, and the normal architecture was altered, with irregular morphological changes evident. After drug intervention, the pathological damages in both cardiac and lung tissues were remarkably reduced. The cardiac tissue structure showed signs of recovery, and the disordered state was alleviated. In the lung tissue, the morphology became relatively more regular. (**C**) Cardiac histopathology scores. (**D**) Lung histopathology scores. Scale bar, 200 μm. Data are presented as mean ± standard deviation (*n* = 3). * indicates significant differences compared with the NS group; # represents comparison between the venom group and the drug-treated group (*** *p* ≤ 0.001 and ### *p* ≤ 0.001).

Compared with the normal saline group (NS), the venom-treated group exhibited disorganized myocardial tissue structure in mice, with abnormal cellular architecture observed in localized areas (Figure 7A), indicating venom-induced cardiac tissue damage. Following drug intervention, the cardiac tissue showed structural recovery with reduced disorganization. From a pathological analysis perspective, this provides an explanation for the improvements in biochemical markers such as CK and LDH after drug treatment. Histological staining of lung tissues revealed that venom administration caused significant morphological alterations, including irregular architecture, evident hemorrhage, loss of alveolar structure, and nodular formations (Figure 7B). In contrast, the drug-treated group displayed relatively regular pulmonary morphology, demonstrating the drug’s ameliorative effect on venom-induced lung injury. These findings collectively demonstrate that the drug effectively alleviates pathological damage to cardiac and pulmonary tissues caused by snake venom, providing histopathological evidence for its therapeutic role in anti-snake venom injury therapy.

## 3. Discussion

Snakebite envenoming remains a critical health threat for low-income populations in tropical regions globally [1,32]. Current antivenom therapies exhibit several limitations that hinder their clinical application, particularly in rural, resource-limited settings where victims typically require 5–9 h to reach medical facilities, significantly delaying treatment [33,34]. This necessitates novel therapeutic approaches to address the shortcomings of serum-based therapies. This study investigates two small-molecule compounds, Rhamnetin and RXP03, for their inhibitory effects on venom toxicity. By establishing animal injury models through venom inoculation, we systematically characterized venom-induced oxidative damage. Our findings demonstrate that the combined application of Rhamnetin and RXP03 significantly inhibits the enzymatic activities of two core venom components, PLA_2_ and SVMP. In murine models, this combination effectively mitigated venom-induced local tissue damage, systemic organ dysfunction, coagulation abnormalities, and improved survival rates. These conclusions align with previous reports on SaMPI/SaPLIγ and varespladib/marimastat combination therapies. Our results provide critical experimental evidence for developing novel broad-spectrum small molecule antivenoms and highlight the potential advantages of multi-target combination strategies. Moreover, the oxidative damage data generated in this study offers valuable insights into venom-organism interactions.

PLA_2_ and SVMP constitute core toxic components in Viperidae and Elapidae venoms [20,21,22,24], mediating diverse pathological effects including hemolysis, myonecrosis, hemorrhage, and coagulopathy. PLA_2_ directly targets membrane components, while SVMP hydrolyzes extracellular matrix proteins, synergistically inducing severe hemorrhagic and hemolytic manifestations [35,36,37]. Rhamnetin, a flavonoid secondary metabolite with multi-pharmacological properties, exerts PLA_2_ inhibition through direct antagonism [27]. In vitro enzymatic assays confirmed its dose-dependent suppression of PLA_2_ activity across multiple venoms, consistent with prior findings [28]. RXP03, a broad-spectrum metalloproteinase inhibitor with established efficacy against MMP-8, demonstrated potent SVMP inhibition [29]. The combination therapy significantly attenuated subcutaneous hemorrhage induced by diverse venoms, strongly supporting Casewell’s proposed “cocktail therapy” strategy [10].

Snake envenomation induces complex pathologies including coagulopathy, local hemorrhage/edema, myonecrosis, and multi-organ failure [38,39,40]. Our systematic characterization of venom-induced damage in animal models revealed severe muscular/visceral injury and coagulopathy. Tissue damage and organ failure arise from both direct enzymatic action (PLA_2_/SVMP) and secondary inflammatory/oxidative cascades [26,41,42]. PLA_2_-mediated membrane hydrolysis releases lysolecithins and free fatty acids that trigger inflammatory responses [43,44], while SVMP degradation of type IV collagen compromises vascular integrity [42,45]. Subsequent hemoglobin/myoglobin release amplifies innate immune activation [46]. In this study, envenomed mice exhibited muscular hemorrhage, elevated apoptotic and inflammatory markers, inflammatory cell infiltration, partial necrosis, reduced tensile strength, and oxidative imbalance. The Rhamnetin/RXP03 combination significantly alleviated these effects, restoring antioxidant parameters and normalizing inflammatory/apoptotic protein expression.

Venom can induce not only localized tissue damage but also inflict severe systemic harm to the entire organism [41]. As the principal metabolic and detoxification organs, the liver and kidneys serve as primary target organs for toxic agents. This pathological manifestation has been corroborated by autopsy findings from victims of D. acutus (hundred-pace pit viper) envenomation [31]. Furthermore, cardiac and pulmonary tissues, being central to circulatory system physiology with their substantial blood perfusion volumes, demonstrate heightened susceptibility to injury through both secondary pathophysiological mechanisms and direct toxic assault from venom components. In this study, venom-challenged mice exhibited marked elevation of urinary protein, LDH, SCr, and myoglobin levels, accompanied by an abnormal AST/ALT ratio, indicative of systemic metabolic and circulatory dysfunction induced by venom toxicity. Histopathological analysis revealed characteristic tissue damage patterns including hepatic hemorrhage with cellular necrosis, loss of renal tubular brush borders, cardiac inflammatory cell infiltration, and pulmonary hemorrhage. These pathomorphological alterations are likely attributable to the direct cytotoxic effects of SVMP and PLA_2_. Mechanistically, SVMP mediates extracellular matrix (ECM) proteolysis, thereby potentiating inflammatory cascades through ECM degradation products [47]. PLA_2_ demonstrates synergistic toxicity through complementary mechanisms of action. Notably, the envenomed mice manifested severe coagulopathy characterized by hypofibrinogenemia (marked depletion of fibrinogen) and prolonged coagulation parameters. The dual action of SVMP and PLA_2_ disrupts hemostatic equilibrium through coordinated interference with coagulation/fibrinolytic systems and platelet function, while simultaneously inducing erythrocyte membrane destabilization, collectively exacerbating systemic pathophysiological deterioration [48]. Therapeutic intervention with Rhamnetin and RXP03 significantly mitigated venom-induced systemic complications and improved survival outcomes. Histopathological evaluation demonstrated substantial attenuation of structural damage in hepatic, renal, cardiac, and pulmonary tissues in treated cohorts, with findings correlating with normalized serum biochemical markers (ALT, AST, SCr, etc.). This investigation confirms that pharmacological inhibition of SVMP and PLA_2_ activities represents an effective therapeutic strategy for snakebite envenomation, aligning with previous findings [10,41]. However, incomplete tissue recovery post-treatment may suggest potential involvement of auxiliary venom components, highlighting the necessity for optimized multidrug regimens or refined therapeutic protocols to achieve comprehensive tissue repair.

Although antivenoms remain the clinical standard, their limitations in specificity, storage, and accessibility impede their utility in resource-poor regions [5,6,9]. This study provides pharmacological evidence supporting alternative approaches and reinforces the reliability of cocktail therapies. Notably, the broad-spectrum efficacy of this dual-target strategy could reduce dependence on species-specific antivenoms, aligning with WHO’s goal to halve snakebite mortality by 2030 [4]. However, this study was limited to murine models and did not evaluate the efficacy or safety of the drugs in large mammals such as primates. Also, the administration method involving pre-incubation of venom with drugs prior to injection diverges from real-world envenomation scenarios where venom has already been disseminated. Further investigations are required to explore therapeutic windows and the effects of delayed drug administration. The rapid degradation of extracellular matrix (ECM) components by venom metalloproteinases (SVMPs) poses a critical challenge for sustained drug delivery in envenomed tissues [16,49,50]. MPs not only compromise tissue integrity but also alter local microenvironments, potentially hindering drug penetration and retention [51]. Future studies should explore new delivery systems to overcome these obstacles, as proposed in recent advances for toxin-targeted therapies [49,52]. Moreover, there are significant differences in the composition and concentration of venom components among different snake species (especially in the SVMP/PLA_2_ subtypes of viperine and colubrine species), which may affect the clinical treatment outcome [53].

In conclusion, this study is the first to demonstrate the therapeutic potential of the Rhamnetin–RXP03 combination against snake venoms, laying a theoretical foundation for developing cost-effective, broad-spectrum, and storage-stable treatments. Successful clinical translation of this strategy could transform snakebite management, particularly in underserved regions with limited healthcare access. Antitoxin serum is the most effective treatment method. However, other treatment options, such as small molecule drugs, can serve as a supplement to antitoxin serum or help delay the treatment time for patients [22,54,55].

## 4. Conclusions

Snakebite poses significant health risks, and current antivenom therapies have limitations. This study evaluated the antivenom efficacy of small-molecule inhibitors Rhamnetin and RXP03. The combined administration of these two drugs effectively inhibited key venom core toxins (PLA_2_ and SVMP), markedly alleviated venom-induced local tissue damage, systemic organ dysfunction, and coagulation abnormalities, and improved survival rates in mice. Although further exploration of their clinical translational potential and safety is needed, this dual-target strategy demonstrated broad-spectrum potential, providing a critical foundation for the development of next-generation antivenom therapeutics.

## 5. Materials and Methods

### 5.1. The Snake Venom and Main Reagents

*D. acutus*, *P. mucrosquamatus*, *G. blomhoffi*, *N. atra*, and *T. stejnegeri* were provided by Huangshan Snake Farm (Jingdezhen City, Jiangxi Province, China). Select the snake species for the research from the snake farm, with five of each species. Encourage the snake venom to penetrate through the membrane covered with a cellulose film and enter a 50 mL centrifuge tube. Then, combine the venom obtained from the same species of snakes from different individuals, freeze-dry it, and store it at −20 °C before use. Both Rhamnetin (catalogue: HY-N7036, ≥95% by HPLC) and RXP03 (catalogue: HY-162038, ≥95% by HPLC) were purchased from MedChemExpress^®^ (Wuhan, Hubei Province, China). Azocasein and 4-nitro-3-octanoyloxybenzoic acid (NOB) were purchased from Macklin^®^ (Shanghai, China) and Aladdin^®^ (Shanghai, China), respectively.

### 5.2. PLA_2_ Activity

According to the method described by Haberman, fresh sterile egg yolks were mixed with saline (1:3 ratio) and then centrifuged at 4500 rpm for 10 min, retaining the supernatant as substrate. A 3% agarose solution was configured with NaAC buffer and heated until the agarose was completely dissolved. After cooling to 55 °C, 4% substrate and 2% CaCl were added. The mixture was poured into a petri dish, cooled until solidified, and 6 holes were punched. In this study, venom (10 µg) was pre-incubated for 30 min at 37 °C with Rhamnetin in 1:0, 1:0.1, 1:0.2, 1:0.4, 1:0.8, and 0:1 (venom/Rhamnetin, *w*/*w*) ratios at a final volume of 100 µL. The incubated solution was added sequentially to the wells of the plate, with saline added to the last well. The plates were incubated in a 37 °C thermostat for 12 h, after which the size of the transparent circles was observed [56].

In reference to previous literature with some adaptations [57], the previously prepared solution of venom and Rhamnetin after incubation was pre-incubated with 100 µL NOB (1 µg) solution for 30 min (37 °C). After the incubation, 100 µL of 2.5% Triton X-100 was added, mixed well, and the absorbance was measured at 334 nm. The results were expressed as inhibitory efficiency of Rhamnetin in relation to the control, in which only venom was incubated, and were reported as the mean ± standard error of the mean (*n* = 3).

### 5.3. SVMP Activity

The efficiency of RXP03 in inhibiting protease activity was evaluated colorimetrically through enzymatic degradation of azocasein, as previously described [58]. In our study, venom (10 µg) was pre-incubated for 30 min at 37 °C with RXP03 in 1:0, 1:0.1, 1:0.2, 1:0.4, 1:0.8, and 0:1 (venom:RXP03, *w*/*w*) ratios at a final volume of 100 µL. Then 100 µL of casein was added to each group, and incubation continued for 90 min at 37 °C. The blank group was replaced with an equal volume of 50 mM Tris-HCl. After incubation, the reaction was terminated by adding 100 µL of 5% trichloroacetic acid at 25 °C for 30 min, followed by centrifugation at 12,000 rpm/min for 10 min. 100 µL of the supernatant was mixed with an equal volume of 0.5 M NaOH, and the absorbance was measured at 440 nm using a microplate reader. The proteolytic activity was calculated as the percentage of degradation products of the formed azocasein. The results were expressed as inhibitory efficiency of RXP03 in relation to the control and are reported as the mean ± standard error of the mean (*n* = 3).

### 5.4. Animals Model and Ethics

Kunming mice (male, approximately 25–35 g, 6–8 weeks of age) were obtained from the Animal Center of Nanchang University (Nanchang, Jiangxi Province, China). According to procedures previously described [41], each experimental group comprised three mice, with a total of twelve groups in the study. (a) Normal saline (NS); (b) Drug (0.1 µg Rhamnetin and 0.15 µg RXP03); (c, e, g, i, k) treatment with *D. acutus* (DA group), *P. mucrosquamatus* (PM group), *G. blomhoffi* (GB group), *N. atra* (NA group), *T. stejnegeri* (TS group) venom; (d, f, h, j, l) the same as c, e, g, i, and k, but the venom was pre-incubated with these two drugs. All groups of compounds were prepared in saline (total volume 100 µL) and incubated at 37 °C for 10 min before injection. All animal experiments were conducted in accordance with the guidelines for animal experiments at Nanchang University and protocols approved by the Nanchang University Animal Ethics Committee (Ethics code: NDSYDWLL-202131).

### 5.5. Hemorrhagic Activity

Minor modifications were made to the protocol presented in the literature to measure the hemorrhagic activity of the drug inhibiting the venom [56]. The hemorrhagic concentration of venom (20 µg/animal) was previously determined. For this test, the mice received a subcutaneous injection (s.c.) of the previously incubated solution. After 3 h, the animals were euthanized, and the skin tissue was photographed.

### 5.6. Myotoxic Activity

Myotoxic activity was evaluated as described in the literature [25], with minor modifications. The dose was 0.2-fold LD50 of *D. acutus* (8.12 mg/kg), *P. mucrosquamatus* (6.85 mg/kg), *G. blomhoffi* (7.42 mg/kg), *N. atra* (0.67 mg/kg), and *T. stejnegeri* (1.51 mg/kg) venom. Grouping and incubation were performed as previously mentioned, and then the solutions were injected via musculus gastrocnemius (i.m.) injection. After 12 h, muscle tension was measured, and mice gastrocnemius muscles were collected for Western blot and oxidative stress assays.

### 5.7. Venom-Induced Systemic Toxicity

To assess the inhibition of drugs on systemic toxicity, the protocol proposed in the literature was carried out with minor modifications [41]. The intraperitoneal (i.p.) administration dose was 0.2-fold LD50 of *D. acutus* (2.8 mg/kg), *P. mucrosquamatus* (2.815 mg/kg), *G. blomhoffi* (1.32 mg/kg), *N. atra* (0.074 mg/kg), and *T. stejnegeri* (0.8 mg/kg) venom, based on the above grouping and preincubation. Animal behavioral experiments were performed after 12 h. The trajectories of the mice were recorded over a 5 min period, and the distance traveled was calculated. The mice were euthanized, and the serum was harvested for sero-enzyme assays [59]. Urine was collected for protein content analysis, and heart, lung, liver, and kidney tissues were isolated for hematoxylin–eosin staining.

### 5.8. Ser-Enzyme, Urine Protein, and Oxidative Stress Assays

Commercial kits (Nanjing Jiancheng Biological Engineering Institute^®^, Nanjing, China) were used to analyze previously collected serum, urine proteins, and gastrocnemius muscles. The parameters analyzed included alanine aminotransferase (ALT), aspartate aminotransferase (AST), creatine kinase (CK), lactate dehydrogenase (LDH), myoglobin, free hemoglobin, uric acid (UA), serum creatinine (Scr), urine protein, catalase (CAT), glutathione (GSH), total superoxide dismutase (T-SOD), total antioxidant capacity (T-AOC), reactive oxygen species (ROS), and malondialdehyde (MDA).

### 5.9. Evaluation of Hemostatic Parameters

The measurement of fibrinogen concentration (FIB), prothrombin time (PT), thrombin time (TT), and activated partial thromboplastin time (APTT) were determined using commercial kits (Rayto Life and Analytical Sciences^®^, Wuhan, China) and an automatic coagulation analyzer (Servicebio^®^, Wuhan, China) according to the manufacturer’s protocols.

### 5.10. Western Blot

Proteins were extracted from gastrocnemius muscle tissue using RIPA buffer (1 mM PMSF and protease inhibitor). Denatured protein samples were resolved using sodium dodecyl sulfate-polyacrylamide gels and transferred to polyvinylidene difluoride membranes. The membranes were blocked with blocking buffer for 2 h. The membranes were incubated with Bcl-2, Bax, TNF-α, and IL-6 antibodies (Wanlei Biotechnology^®^, Changchun, China) for 2 h at room temperature, as well as with GAPDH antibody (Proteintech^®^, Wuhan, China) as loading controls. The membranes were then incubated with anti-rabbit IgG-HRP-conjugated or anti-mouse IgG-HRP-conjugated secondary antibodies for 1 h and visualized using enhanced chemiluminescence reagent (UElandy^®^, Nanchang, China). Quantification of protein expression levels was performed using ImageJ software V1.8.0.112.

### 5.11. Histological Analysis

Histopathological analysis was conducted based on previous studies with modifications [41,43]. Tissue samples were fixed in 4% paraformaldehyde buffer for at least 24 h, dehydrated in ethanol, embedded in paraffin, and cut into 5 µm sections. Sections were deparaffinized with xylene and then stained with H&E. Histological scores (grades 0–4) were determined for each section based on prior literature with modifications [60]. Grades 0, normal muscle fiber architecture without any pathological alterations. Grades 1, focal edema and/or inflammatory cell infiltration (occupying < 10% of the microscopic field of view), accompanied by individual muscle fiber disarrangement. Grades 2, multifocal myonecrosis (involving 10–30% of the tissue area) with moderate inflammatory infiltration. Grades 3, diffuse myonecrosis (30–60% tissue involvement), hemorrhagic changes, and loss of striated muscle fiber structure. Grades 4, extensive necrosis (>60% tissue involvement) with myolysis and vacuolization of muscle fibers. Three independent pathologists analyzed and reported each section in a blinded manner.

### 5.12. Statistical Analysis

All results were expressed as the mean ± standard error of the mean (SEM). All data were analyzed by one-way ANOVA followed by Tukey’s test (for multiple-group comparisons) or Student’s *t*-test (for two-group comparisons) using GraphPad Prism software (version 8, USA). *** *p* < 0.001, ** *p* < 0.01, and * *p* < 0.05 were considered statistically significant.

## Data Availability

The original contributions presented in this study are included in the article. Further inquiries can be directed to the corresponding author.
